# People who inject oral morphine favor experimentation with injectable opioid substitution

**DOI:** 10.1186/s12954-023-00866-y

**Published:** 2023-09-12

**Authors:** Célian Bertin, Philémon Dècle, Pierre Chappard, Perrine Roux, Nicolas Authier

**Affiliations:** 1 CHU Clermont-Ferrand, Inserm 1107, Neuro-Dol, Service de Pharmacologie Médicale, Centres Addictovigilance et Pharmacovigilance, Centre Evaluation et Traitement de la Douleur, Université Clermont Auvergne, BP-69, CHU Gabriel Montpied, 58 Rue Montalembert, 63000 Clermont Ferrand, France; 2 Observatoire Français des Médicaments Antalgiques (OFMA), French Monitoring Center for Analgesic Drugs, Clermont-Ferrand, France; 3 Faculté de Médecine, Institut Analgesia, Clermont-Ferrand, France; 4 INSERM, IRD, SESSTIM, Sciences Economiques and Sociales de la Santé and Traitement de l’information Médicale, ISSPAM, Aix Marseille Univ, Marseille, France; 5 Association PsychoACTIF, Marseille, France

**Keywords:** Opioid, Morphine, Substance use disorder, Overdose, Opioid maintenance treatment, Prescription medication misuse, Morphine dependence, Opioid substitution treatment, Injectable substitution treatment

## Abstract

**Background:**

The French Addictovigilance network has observed the existence of the intravenous use of oral morphine capsules among people suffering from opioid use disorders. According to persons who inject morphine, these capsules are easy to dissolve and then inject, giving them the image of an "injectable" opioid substitution treatment (OST). In France, validated OSTs are only available orally, so dissolving morphine capsules represents the only alternative for patients who are not sufficiently relieved by oral forms.

This practice presents risks related to the potential persistence of particles of the oral galenic in the injectable solution, despite its filtration, but also risks—notably of overdose—related to the pharmacological effects of opioids and to variations of the quantities of morphine extracted during the dissolution of the capsules. We conducted an online survey among the people concerned to collect data on their needs and expectations regarding a possible injectable substitution.

**Method:**

An anonymous online survey including all voluntary respondents residing in France and using oral morphine intravenously was conducted in partnership with the Psychoactif harm reduction organization, from 23/03/2020 to 01/04/2021.

**Results:**

The analysis of the 157 exploitable questionnaires showed that 41% of the respondents obtained their drugs only from illegal markets. The others received, regularly or occasionally, medical prescriptions, reimbursed in 84% of cases. For 78% of the respondents, injection was the most frequent route of morphine administration, with 3.8 ± 2 injections per day. 56% of the respondents were receiving an OST, on prescription (79%), monthly (86%), in addition to morphine.

Skenan® capsules were the most frequently used (81%) and 47.2% of the respondents had already experienced injection-related complications. 95% of the respondents were in favor of experimenting with an injectable morphine substitution. Those who never received medical prescriptions were the youngest (< 25 years) respondents, they reported only occasional use of morphine, and always intravenously.

**Conclusion:**

Oral morphine capsules dissolved and injected intravenously are not a safe and sustainable injectable substitution. Respondents wish to be able to benefit from an injectable substitution with a formulation adapted to the intravenous route. The availability of an injectable substitution would facilitate harm reduction and entry into care for the people concerned, particularly the youngest who have never received morphine prescriptions.

**Supplementary Information:**

The online version contains supplementary material available at 10.1186/s12954-023-00866-y.

## Background

Opioid use disorder is a diagnosis introduced in the fifth edition of the Diagnostic and Statistical Manual of Mental Disorders [1], combining the separate diagnoses of dependence and abuse from the previous edition [2, 3]. The diagnosis applies to anyone who maintains their opioid use for more than 12 months, despite negative consequences for themselves and those around them [4]. The initiation of an opioid substitution treatment (OST) is the recommended addiction treatment [5]. The efficacy and tolerance of OSTs are frequently discussed by people treated for opioid use disorder [6], a source of dissatisfaction that is regularly linked to the oral-only formulation of validated OSTs (buprenorphine and methadone).The absence of formulations other than oral ones, particularly injectable forms, can hinder access to care. Indeed, the injectable route may represent a choice or a necessity for some people. Some of them may have developed a behavioral dependence on intravenous injection, or have a preference for this mode of administration, which ensures a better bioavailability, favoring faster delivery to the brain opioid receptors, or the injection of less substance to achieve the desired effect [7, 8]. The possibility of prescribing opioid medications other than those validated may then be considered.

In France, a particular pharmaceutical form of oral morphine, Skenan®, has been identified by the national network of Addictovigilance centers in charge of monitoring, assessing and preventing the risks associated with the use of psychoactive substances, as sometimes prescribed in this context. It is a slow-release capsule, marketed for the treatment of persistent, intense pain or pain that is resistant to other analgesics. It is said to be relatively easy to dissolve and inject [9–11].

In France, the number of persons who inject oral slow-release morphine regularly is estimated between 1075 and 1288 individuals, and 949 for occasional users [6, 12]. Based on field and pharmacoepidemiological studies, the persons concerned by this practice are often precarious, sometimes nomadic in their access to care, and difficult to assess in conventional observational studies [9, 10, 13]. The estimate of 2000 to 2300 individuals is obviously an underestimate since it was based on Health Insurance reimbursement data. These databases include only data on medications delivered to pharmacies and reimbursed by the Health Insurance. Deliveries paid directly by the user to the pharmacy, or purchases made on illegal markets are not recorded in these databases. The characteristics of people who never receive reimbursement for their morphine are currently unknown. Consequently, their expectations regarding the most suitable harm reduction material for this practice, and possible experimentation associated with access to injectable substitution therapy remain unknown.

The purpose of this online survey among people who inject oral forms of morphine intravenously was to collect data on field practices regarding the medications used and their procurement, dissolution and filtration techniques, injection equipment, and their expectations regarding a possible intravenous substitution treatment. Secondly, we assessed the potential characteristics of persons who obtain their morphine exclusively from illicit markets compared to those who receive their morphine from prescriptions dispensed in pharmacies.

## Methods

This survey was conducted in accordance with the Checklist for Reporting Results of Internet E-Surveys (CHERRIES) aimed at improving the quality of surveys conducted on the Internet [14], and was approved by the South-East VI Committee for the Protection of Persons and registered under the ethical notice number 2022/CE25.

### Study design and sample

This open online survey was conducted from 23/03/2020 to 01/04/2021. The questionnaire, available in original language in the (Additional file 1: Fig. S1), was drafted jointly by the Medical Pharmacology Service of the University Hospital of Clermont-Ferrand, and the associative platform "PsychoACTIF". The latter offers a blog and a French-speaking open access forum on harm reduction (HR), which constitutes a space of solidarity for people who use psychoactive products. These sites providing information and recommendations on the prevention of health problems related to the use of psychoactive substances attracted 2.8 million single visitors in 2021, while the forum alone has more than 39,500 members [15, 16].

### Survey and measures

A presentation of the researchers and the objectives of the survey was published by the administration team of the "PsychoACTIF" forum in a topic dedicated to morphine injection (the survey presentation message is available in original language in the Additional file 1: Fig. S2). A link to the online questionnaire was proposed following this presentation. Respondents were free to participate anonymously, spontaneously, and voluntarily in the survey.

The link led to a page repeating the purpose of the survey and asking for the respondent's consent. Once this consent was obtained, the questionnaire began with three mandatory inclusion questions aimed at confirming the use of oral morphine by intravenous route in France (see questions 1 to 3 of the questionnaire in the Additional file 1: Fig. S1).

If the respondent met the inclusion criteria, *i.e.*, residing in France and using oral morphine intravenously, the survey continued by collecting demographic data, information on the morphine used and its supply, the injection preparation methods and multiple substance use patterns, and the occurrence of health complications associated with morphine injection. The survey concluded by collecting respondents' opinions and comments on the possibility of injectable substitution.

Answers to the 32 questions marked with an asterisk in the questionnaire were required to proceed to the next questions. The questionnaire was designed to adapt the questions proposed to the respondent's previous answers. In this way, only questions relevant to previous answers were asked. This adaptability reduced the time required to complete the survey and favored its completion. Once the questions were validated, the respondents were technically unable to correct their previous responses. When an "Other" item was checked, the respondents were offered a free text box to provide clarifications. This free text box was accompanied by an alert telling the respondents not to enter any information that would remove their anonymity.

### Data analysis

The responses were collected using REDCap® software, ensuring electronic data acquisition and security [17]. The REDCap web platform (https://project-redcap.org) possesses the authorization of the French personal data protection authority. In order to guarantee respondent anonymity, no IP addresses and/or tracking cookies were collected for this survey. In order to preserve the anonymity of the respondents as much as possible, it was necessary to manually check for duplicates and/or multiple respondents by comparing the dates and times of connection to the questionnaire and the proximity of the responses provided. In these cases, the questionnaires were excluded from the analyses.

The data were described by numbers and associated percentages for categorical variables and by the mean ± standard deviation [minimum–maximum] for quantitative variables. To evaluate the potential characteristics of persons who obtain their morphine exclusively from illicit markets compared to those who receive all or part of their morphine from prescriptions dispensed in pharmacies, a univariate logistic regression model was performed. In this context, the age variable was recoded to compensate for the small number of respondents in the initial categories, to ensure the validity of the statistical analyses. The associated p-values were computed with their corresponding odds ratios (ORs) and their 95% confidence intervals (95% CI). Then, a multivariate logistic regression analysis was performed to study the potential differences between the two groups. All the variables associated with *p* < 0.25 in the univariate analysis were included in the model and selected in a top-down manner by retaining those allowing the best possible Akaike information criterion to be obtained. The corresponding adjusted ORs (aORs) were calculated with their 95% CIs. All the statistical analyses were conducted using SAS-9.4 software (SAS Institute, USA) and STATA-14.2 (StataCorp, USA).

## Results

### Population description

**Fig. 1:**
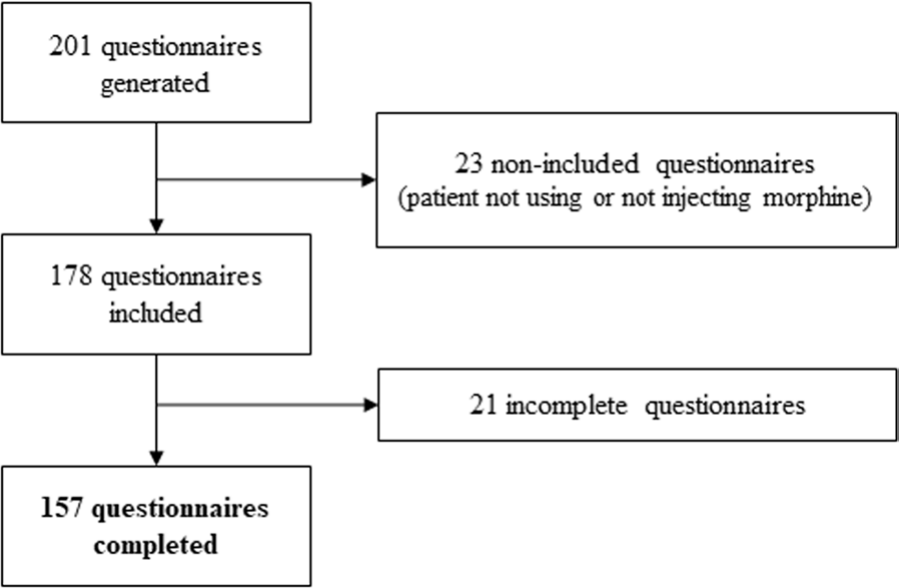
Flowchart of the questionnaires included in the study

Among the 201 forms generated from the link published on the PsychoACTIVE forum, 178 met the inclusion criteria, 157 questionnaires were included in the analysis (21 incomplete questionnaires were excluded) (Fig. 1). Only one duplicate survey was identified, and automatically excluded from the analyses.

The completeness rate (number of usable questionnaires / number of questionnaires meeting the inclusion criteria) was 88.8%.

The responses to the questionnaires are presented in Tables 1, 2 and Additional file 1: Tables S1 and S2.

**Table 1:** Socio-demographic, behavioral and health data of respondents

	Frequency (Number)	Percent
*Gender*		
Male	119	75.8
Female	30	19.1
Do not wish to answer	8	5.1
*Age*		
< 18 years old	11	7.0
18–24 years old	28	17.8
25–29 years old	32	20.4
30–34 years old	22	14.0
35–39 years old	26	16.6
40–45 years old	16	10.2
≥ 46 years old	21	13.4
Do not wish to answer	1	0.6
*Do you have medical prescriptions and pharmacy delivery for this morphine-based medication?*		
Always	51	32.5
Sometimes	26	16.6
Rarely	16	10.2
Never	64	40.8
***(****Frequency missing* = *0)*		
*Are these morphine deliveries reimbursed by the National Health Insurance?*		
Yes	78	83.9
No	15	16.1
***(****Frequency missing* = *64)*		
*Do you inject morphine regularly (several times a week or daily) or occasionally?*		
Regular	93	60.8
Occasional	60	39.2
***(****Frequency missing* = *4)*		
*Is intravenous injection your most common route of administration?*		
Yes	120	78.4
No	33	21.6
***(****Frequency missing* = *6)*		
*Do you ever take any of your morphine by the oral route?*		
No	101	66.0
Yes	52	34.0
*(Frequency missing* = *4)*		
*Which of the following morphine-based medications do you use most often?*		
Skenan	119	81.0
Actiskenan	11	7.5
Morphine hydrochloride (injectable vials)	9	6.1
Other (specify)	5	3.4
*(Frequency missing* = *13)*		
*Do you ever inject substances other than morphine (heroin, cocaine, other…)?*		
Yes	116	80.0
No	29	20.0
*(Frequency missing* = *12)*		
*In addition to these injections, do you take, even occasionally, oral substitution medication?*		
Yes	81	56.3
No	63	43.8
*(Frequency missing* = *13)*		
*Is the oral substitution medication taken occasionally in addition to morphine methadone or buprenorphine?*		
Methadone	60	74.1
Buprenorphine	16	19.8
Both	5	6.2
*(Frequency missing* = *0)*		
*Prescribed or bought on illegal markets?*		
Prescribed only	64	79.0
Prescribed and purchased on illegal markets	9	11.1
Purchased on illegal markets only	8	9.9
*(Frequency missing* = *0)*		
*Are these oral substitution medication prescriptions regular (approximately monthly)?*		
Yes	63	86.3
No	10	13.7
*(Frequency missing* = *0)*		
*Have you ever experienced complications related to injections or morphine? (Overdose, injection site infections, systemic infections: hepatitis C, AIDS, endocarditis, spondylodiscitis, *etc*.)*		
No	76	52.8
Yes	68	47.2
*(Frequency missing* = *13)*

**Table 2:** Expectations, benefits, and user-reported advantages of an injectable morphine compared to an oral formulation

	Frequency (Number)	Percent
*Would the availability of injectable morphine, for example in the form of vials of different dosages, be an alternative that you would accept?*		
Yes	132	95.0
No	7	5.0
*(Frequency missing* = *18)*		
*Would the weekly pharmacy delivery of morphine vials for home self-injection be acceptable in your opinion?*		
Yes	129	92.8
No	10	7.2
*(Frequency missing* = *18)*		
*In your opinion, what would be the benefit of making injectable morphine available?* *(You may check multiple answers)*		
It would allow me to inject a cleaner product, in order to limit the risks of complications	123	77.9
I would no longer need to buy morphine and/or heroin on illegal markets	98	62.0
I could receive an injectable substitution covered by the National Health Insurance	84	53.2
I could formalize a behavior that I already practice	78	49.4
It would make available an injectable substitution in case of intolerance to oral substitution medication	58	36.7
It would allow me to benefit from a transitory injectable substitution before switching to an oral treatment	38	24.1
Other (specify)	12	7.6
*Could you tell us in a few words the advantages you expect from an injectable form compared to the capsules or tablets currently used? It would:*		
Reduce the risks associated with the excipients of the oral galenic	58	47.5
Reduce the risk of infection (hygiene, sterile product without contamination)	52	42.6
Simplify preparation and reduce handling through using a galenic adapted to injection	45	36.9
Improve knowledge, adaptability and reproducibility of the injected dose	20	16.4
Guarantee the pharmaceutical quality of the injected substance	18	14.8
Allow entering a validated care system, moving away from illicit markets, stopping the misuse of oral morphine	6	4.9
Provide an alternative in case of insufficient efficiency of oral OST	5	4.1
Obtain a more rapid psychoactive effect (maintenance of a shoot effect)	4	3.3
Reduce the injection of other substances by better reducing the craving to inject	3	2.5
Reduce stigma, recognize and manage injection as a behavioral addiction	2	1.6
Reduce the financial cost by having injections covered by the National Health Insurance	2	1.6

The respondents were predominantly male, between 18 and 29 years of age (Table 1).

**Fig. 2:**
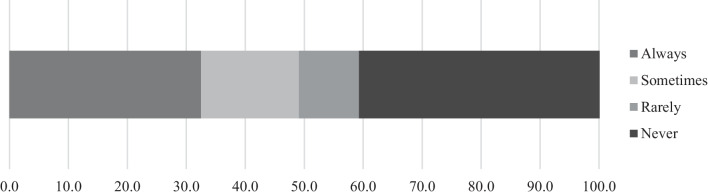
Proportion of respondents with a medical prescription and pharmacy delivery of their opioid medication

The proportion of respondents receiving systematic or occasional dispensing from a pharmacy was similar to the proportion who obtained their supplies only from illicit markets (Fig. 2). When prescribed, the cost of morphine was most often covered by the National Health Insurance (Table 2).

**Fig. 3:**
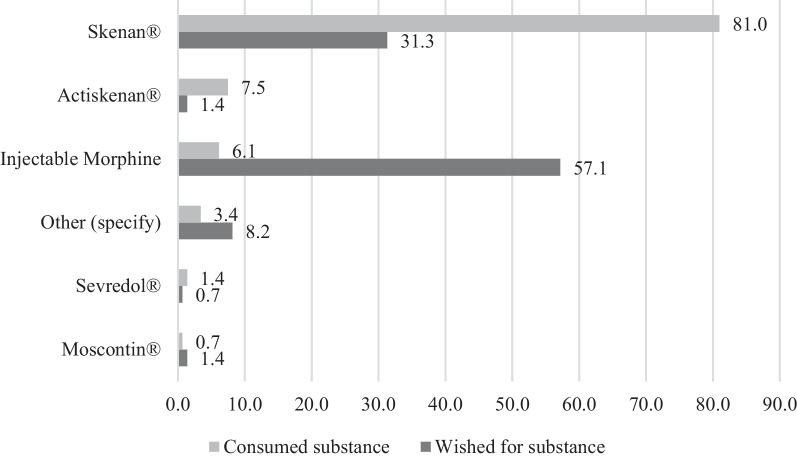
Morphine injected by users compared to their expectations

Most of the responders were regular intravenous Skenan users (Fig. 3), corresponding to their main route of administration. The average number of daily injections was 3.8 ± 1.9 (Min–Max: 1–10). Only one third of the respondents indicated that they administered part of their morphine orally.

Most of the respondents wanted morphine injection vials, rather than diverting oral Skenan capsules. Several comments indicated the respondents' lack of awareness of the availability of injectable morphine at the community pharmacy (Table 2).

Injections were most often performed with 2 ml syringes equipped with orange needles (25 gauge), which also corresponded to what would be the choice of the respondents if it were left to them (Additional file 1: Table S1, Additional file 1: Figs. S3 and S4). The dissolution of oral morphine was mostly done with sterile water, at room temperature, without boiling before or after dissolution. The filtration of the solution was divided between the use of a "Spinning top" filter (29.7%), the Stérifilt® (24.8%), or the cotton provided in the Stéribox® (24.8%) (pictures of the different filters are available in Additional file 1: Fig. S5). More rarely, respondents used a simple cigarette filter (13.1%), or did not filter their morphine solution (4.8%).

Four out of five respondents reported injecting substances other than morphine, most often heroin and cocaine, whether based or not (Table 1). Half of the respondents reported receiving a validated OST on a regular or occasional basis, most often prescribed monthly. Methadone was prescribed in three quarters of these cases.

Nearly half of the respondents reported having experienced complications related to intravenous oral morphine injections (47.2%). One out of two of these were local bacterial infections, and a quarter of the respondents had experienced overdose or hepatitis C virus (HCV) seroconversion (Additional file 1: Table S2).

### Expectations about injectable substitution

Most respondents were in favor of a possible substitution by injectable morphine (95.0%), delivered weekly in pharmacies (Table 2). The availability of a suitable dosage form for injection to reduce the risk of complications was mentioned in more than three quarters of the testimonies. The prospect of abandoning purchases from illegal markets, initiating an approach to care covered by the National Health Insurance, and formalizing a practice already in use was mentioned by more than half of the respondents. One third of the respondents mentioned the interest of an injectable substitution for persons who are intolerant to validated OST. A quarter of the comments mentioned the relevance of injectable substitution before a possible transition to a validated OST.

The analysis of expectations regarding an injectable OST (Table 2) showed that half of the respondents wanted to make their intravenous use of morphine safer, by reducing the risks linked to excipients, through a suitable pharmaceutical quality formulation. They also mentioned simplified handling made possible by a formulation adapted to the injectable route, favoring the reduction of infectious risks. Finally, one out of six respondents mentioned the interest of better knowledge, and the adaptability and reproducibility of self-administered doses made possible by the delivery of an injectable substitution validated for this use.

Respondents who were reluctant about the idea of an injectable substitution (5.0%) mentioned the risk of breaking glass vials of morphine, the persistence of infectious risks associated with injection and the possibility of waiting until the time of self-administration to choose between oral or intravenous routes of administration. One respondent also mentioned his or her attachment to the ritual of dissolving oral morphine capsules.

### Characteristics of persons who obtain their morphine exclusively from illicit markets compared to others

**Fig. 4:**
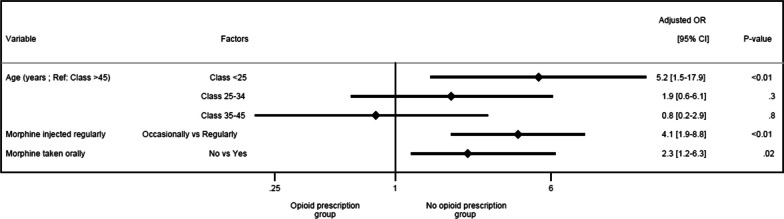
Characteristics of persons who obtain their morphine exclusively from illicit markets compared to those who receive all or part of their morphine from prescriptions dispensed in pharmacies in the multivariate analysis. *OR* Odds ratio

The results of univariate analysis comparing people who never receive a prescription for their morphine to those who receive it (sometimes or regularly) are available in Additional file 1: Table S3. The multivariate model included all the variables associated with *p* < 0.25 in the univariate analysis (Additional file 1: Table 3). The graphical representation of the most efficient multivariate logistic regression model obtained corresponds to the forest plot in Fig. 4. The area under the curve for the multivariate model was equal to 0.76 (Additional file 1: Fig. S6). This model showed that those who purchase morphine exclusively from illicit markets compared with those who obtain their morphine by prescription are younger (*p* < 0.01), especially young adults (< 25 years) (aOR = 5.2 [1.5–17.9] *versus* > 45 years). They do not regularly inject morphine (*p* < 0.01 (aOR = 4.1 [1.9–8.8]), with no reported oral administration (*p* = 0.02 (aOR = 2.3 [1.2–6.3]).

**Table 3:** Results of multivariate analysis comparing people who never receive a prescription for their morphine to those who receive it regularly or not

Associated factors	Ajusted OR	95%CI	*P* (Wald)
*Age*			< .01
< 25 *Versus* > 45 years	5.2	[1.5–17.9]	
25–34 *Versus* > 45 years	1.9	[0.6–6.1]	
35–45 *Versus* > 45 years	0.8	[0.2–2.9]	
*Do you inject morphine regularly (several times a week or daily) or occasionally?*			< .01
Occasionally *versus* regularly	4.1	[1.9–8.8]	
*Do you ever take any of your morphine by oral route?*			0.02
No *Versus* Yes	2.3	[1.2–6.3]

## Discussion

These results provide new information about the prevalence of intravenous oral morphine injection, notably by showing a comparison between the number of respondents who never receive prescriptions and those who receive them (regularly or not). This is an important finding because it suggests that approximately 40% of the persons who inject morphine (PWIM) are omitted by pharmacoepidemiological studies that provide the main known data on this topic. As these studies are based on healthcare reimbursement data, they do not provide any information on purchases made on illicit markets, which escape the healthcare system. This survey provides new information about the practices and expectations of PWIM, particularly concerning their wish to be able to benefit from a substitution by injectable morphine.

Our findings helped us to better understand the profile of PWIM who never have access to a prescription. Compared to respondents who obtain their morphine by prescription, those who purchased their morphine exclusively from illicit markets were the youngest, they used morphine exclusively by injection, but were irregular users. This irregular use of morphine may be surprising, as it contrasts with its exclusively intravenous administration. It can be seen as a one-off search for opioid effects, but we cannot ignore the hypothesis that it could be a search for an injectable substitution. Whatever the case, this practice is all the more worrying among young adults because of their described increased risk behaviors, including overdose, and their hindered access to adapted care [18–21]. Added to the fact that young adults suffering from a substance use disorder are more often subject to comorbidities [18, 20], it therefore seems more important to be able to offer them an injectable substitution treatment, which would attract them to care and initiate a dialogue about injecting and HR. 

A potential explanation for the exclusive supply of these young adults on illicit markets may also lie in the medical profession's apprehension about prescribing an opioid known to be frequently diverted for the injectable route in an opioid use disorder context [9, 10, 13, 22]. This apprehension could be reduced by making it possible to prescribe an injectable opioid substitution, within a clear legal framework, and improving the education of health professionals regarding the needs of PWIM, particularly young adults. As one study of injectable buprenorphine showed, the people most in favor of injectable treatment were those who never received prescriptions for their substance use disorder [23]. The availability of an injectable substitution would be an ideal entry point for the management of opioid use disorder and appropriate HR measures [23, 24]. As young adults appear to be the most precarious people concerned, and therefore the least able to finance their care, it is necessary to facilitate their access to OST, including injectable forms, covered by the national health system [18, 19, 25].

The use of a pharmaceutical specialty, Skenan, predominated among the respondents. This specific choice seemed to be essentially based on practical and galenic considerations. The morphine microbeads contained in the Skenan capsule, allowing slow release, are reputed to be easier to dissolve after crushing than the other oral morphine specialties, marketed in tablet form. The latter may also contain more talc and silica, which are insoluble, not eliminated by the body, and difficult to filter. These substances make injection painful and increase the risks of thromboembolic complications and pneumoconiosis, which can evolve into fibrosis and even pulmonary hypertension [26–28]. Despite a similar formulation, Actiskenan®, an immediate-release form of morphine, has been described by intravenous morphine users as difficult to filter, painful to inject, and it also produces fewer psychoactive effects due to its lower morphine dosages [13, 29].

The analysis of the complications described by the respondents illustrates the progress that can be made in preventing the risks linked to the substance (opioid overdoses), the injection of the oral form (thrombosis, pulmonary embolism), and intravenous injection (human immunodeficiency virus [HIV], HCV, venitis, local and systemic bacterial infection). Each of these risks could be reduced through the implementation of specific HR guidelines.

Overdoses could be prevented by making naloxone (opioid antagonist) rescue kits routinely available to opioid users. It allows reversing the overdose and reducing mortality while waiting for emergency services to intervene. This is particularly important as an increased risk of overdose has been demonstrated in morphine users compared to methadone and buprenorphine users [6, 12]. Complications related to the use of an oral formulation for intravenous injection can be significantly reduced. On the one hand, by facilitating access to sterile, single-use injection equipment including in particular HR filters whose membranes have narrow pores (0.22 µm), which seems particularly relevant in a context where one respondent out of five reports not filtering his or her injectable solution or using only a cigarette filter whose filtration quality is unknown. On the other hand, by making available a formulation adapted to self-injection behaviors while maintaining prevention and treatment objectives. The unavailability of an intravenous OST constitutes a lacuna in the therapeutic arsenal of addictology. This deficiency leaves at least several thousand users with no other option than to inject an imprecise dose of an impure solution of morphine several times a day over the long term.

This gap in the addictological pharmacopoeia was clearly reflected in the comments made by the respondents, who mentioned the relevance of making a validated self-injectable substitution treatment available. This would make it possible to secure their practice, and to offer them early access to a care pathway which would enable them to move away from illicit markets. The availability of an injectable substitution using an adapted galenic would offer PWIM the assurance of self-administering a reproducible dose of opioid. In a recent study, we have shown the great variability of morphine quantities in solution after the dissolution of morphine capsules, according to preparation and filtration conditions [30]. In a substitution context, the stability of self-administered doses is necessary for the adequate control of OUD and avoiding the occurrence of withdrawal symptoms.

Injectable substitution treatment can easily be integrated into existing care systems, since most of the people concerned are also receiving addiction care, during which they are prescribed an oral OST such as methadone. It seems justifiable to recognize the co-prescription of two opioids for substitution purposes, but with different routes of administration, as a valid form of care for the minority of patients who inject opioids intravenously and remain inadequately relieved by oral OST. This co-prescribing of opioids for substitution may be a necessary transitional step, or even a longer-term therapeutic perspective, in the management of certain treatment-refractory opioid use disorders. This therapeutic framework would be similar to that practiced in other countries (including Switzerland, Netherlands, Germany, Denmark, and Canada) with diacetylmorphine and/or hydromorphone-assisted treatment [31–34]. In France, although unofficial, this practice is widely known and even recognized, given the proportion of users whose oral morphine is delivered on prescription and reimbursed by the National Health Insurance. It is remarkable that this practice was even the subject of a parliamentary briefing note, now obsolete, without any further legislative procedure [35]. 

Beyond ensuring that everyone has dignified living conditions with access to basic hygiene, the prevention of injection-related complications would be improved by better informing users about the principles of practicing sterile injection. The availability of a sufficient quantity of HR equipment would encourage its single use for each injection, without sharing between users. The recycling of soiled equipment after injection would limit any risk of accidental contamination and its abandonment in public spaces.

In addition to the availability of injectable opioid substitution, which now appears to be an unavoidable need, the safest setting for dispensing and administration remains to be defined. The respondents were in favor of the weekly pharmacy dispensing of injectable morphine. Perhaps this care setting would not be suitable for some users, particularly the most vulnerable ones, as they do not always have medical coverage that allows their treatment to be reimbursed by the National Health Insurance.

In this context, the care framework proposed by drug consumption rooms seems appropriate and safe [36, 37], offering a harm reduction environment with the supervised self-administration of substances, equipment adapted to practice and a recycling circuit for used equipment. The multidiciplinarity of the care framework proposed in these structures would favor entry into a comprehensive care pathway, including screening and the management of somatic and psychic comorbidities, social precariousness, etc., in parallel to the treatment of substance use disorder using with adapted galenic formulations. These structures have also been shown to reduce systemic viral infections (HIV and HCV), skin complications due to injections, non-fatal overdoses, and emergency room visits [38]. However, care must be taken to ensure that the care framework is sufficiently non-stigmatizing, flexible and respectful of confidentiality to avoid a coercive and restrictive dimension that would undermine its attractiveness and effectiveness, especially in young adults [18, 20, 23, 24, 39].

Despite the limited size of the survey, some demographic data, including the proportion of males among the respondents and their average age, as well as their use practices, particularly their strong preference for Skenan, were consistent with the literature [6, 9, 10, 13]. These data make it possible to confirm the validity of the results obtained, without eliminating certain biases inherent to the methodology chosen.

The choice of an online survey allowed the collection of practices and expectations of users who are outside any care network and usually excluded from conventional research, including people who buy their morphine exclusively on illicit markets, without ever receiving medical prescriptions delivered in pharmacies.

The choice of an online survey has the downside of requiring familiarity with and access to computer tools, frequenting the PsychoACTIF forum, and limiting the collection and interpretability of open-ended responses. These limitations could be corrected by repeating the collection of the practices and expectations of the persons concerned in face-to-face interviews in harm reduction centers for persons who use drugs, and in drug consumption rooms.

## Conclusions

Oral morphine capsules dissolved and injected intravenously cannot represent a safe and sustainable injectable substitution. The respondents wanted to benefit from an injectable substitution with a formulation adapted to the intravenous route. An injectable substitution would make it possible to guide the people concerned towards care, particularly the youngest who never receive morphine prescriptions. In this context, it would also seem justified to recognize the co-prescription of two opioids for substitution purposes, but with different routes of administration, as a valid form of care.

### Supplementary Information

**Additional file 1. **Supplementary file.

## Data Availability

The database is hosted by the REDCap web platform and available upon request from the corresponding author.

## Abbreviations

HCV: Hepatitis C virus
HIV: Human immunodeficiency virus
HR: Harm reduction
OST: Opioid substitution treatment
PWIM: Person who injects morphine
