# Supplementary material for: People who inject oral morphine favor experimentation with injectable opioid substitution
Source: Harm Reduct J. 2023 Sep 12;20:130. doi: 10.1186/s12954-023-00866-y (PMC10496178; doi:10.1186/s12954-023-00866-y)
Supplement: Supplementary file 1 — Additional file 1. Supplementary file. [file 12954_2023_866_MOESM1_ESM.docx]

**People who inject oral morphine favor experimentation with injectable opioid substitution**

Célian Bertin ^1,2,3*^, Philémon Dècle ^4^, Pierre Chappard ^5^, Perrine Roux ^4^, Nicolas Authier ^1,2,3^

^1^ Université Clermont Auvergne, CHU Clermont-Ferrand, Inserm 1107, Neuro-Dol, Service de Pharmacologie médicale, Centres Addictovigilance et Pharmacovigilance, Centre Evaluation et Traitement de la Douleur, Clermont Ferrand, France

^2^ Observatoire Français des Médicaments Antalgiques (OFMA) / French monitoring center for analgesic drugs, Clermont-Ferrand, France.

^3^ Institut Analgesia, Faculté de Médecine, Clermont-Ferrand, France

^4^ Aix Marseille Univ, INSERM, IRD, SESSTIM, Sciences Economiques & Sociales de la Santé & Traitement de l'Information Médicale, ISSPAM, Marseille, France.

^5^ Président de l’association PsychoACTIF

*** Corresponding author:**

Célian Bertin

Service de Pharmacologie Médicale, BP-69, CHU Gabriel Montpied

58 Rue Montalembert, 63000 Clermont-Ferrand, France

Mail address: cbertin[AT]chu-clermontferrand[DOT]fr

ORCID: 0000-0002-2393-706

Keywords: Opioid, morphine, substance use disorder, overdose, opioid maintenance treatment, injection drug user, prescription medication misuse, morphine dependence


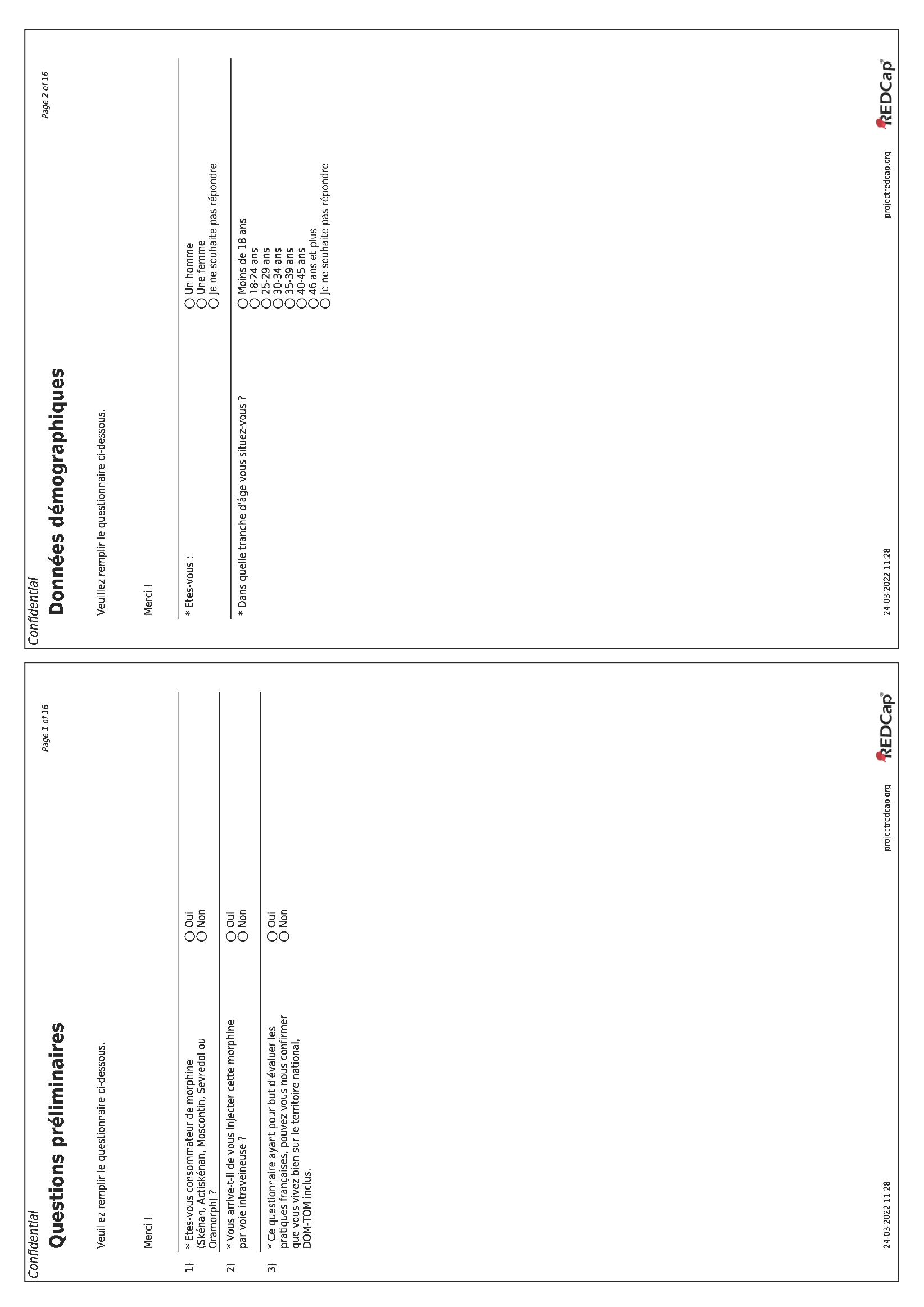


**Figure S1.** Full copy of the online questionnaire, in original language


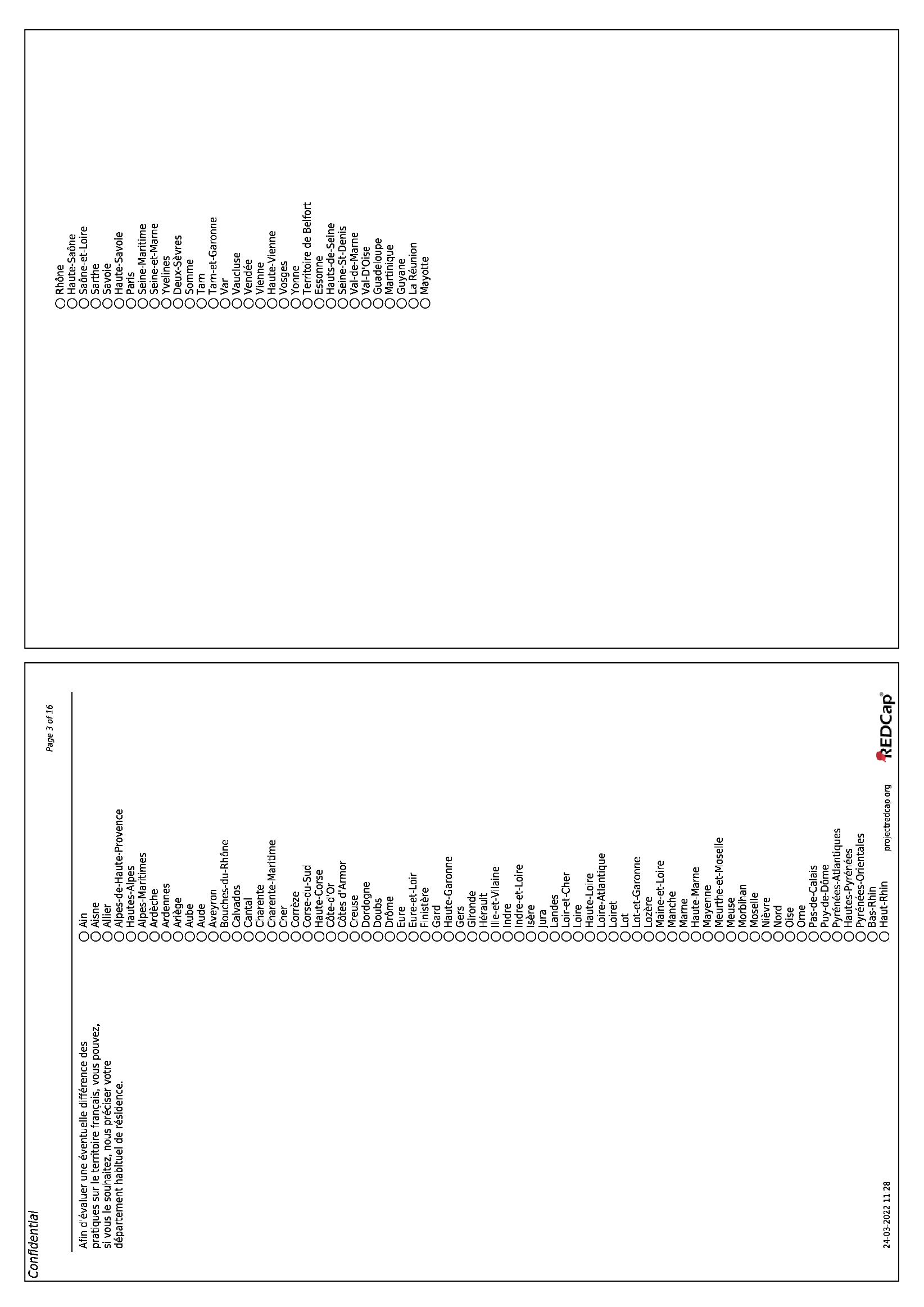


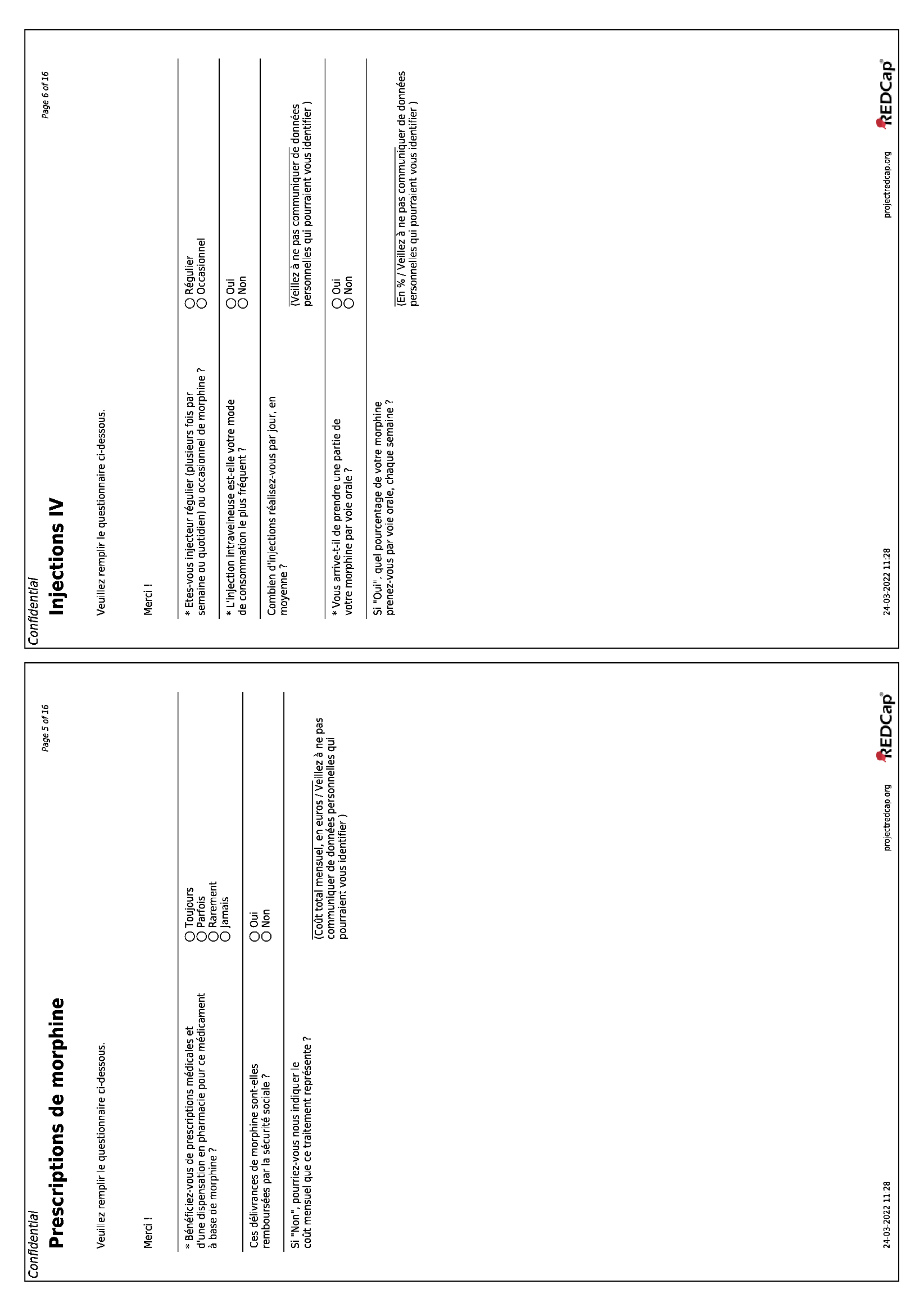


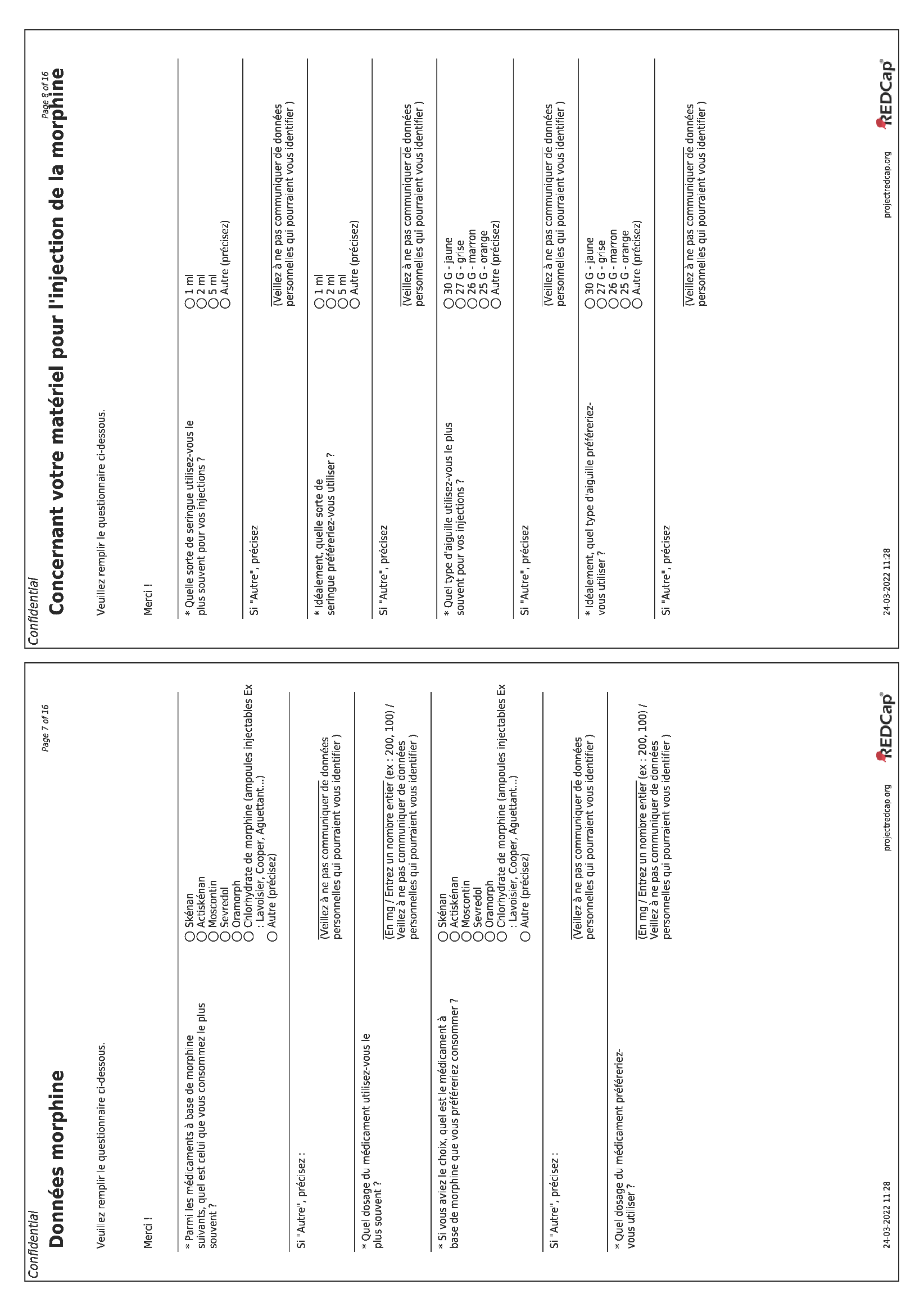


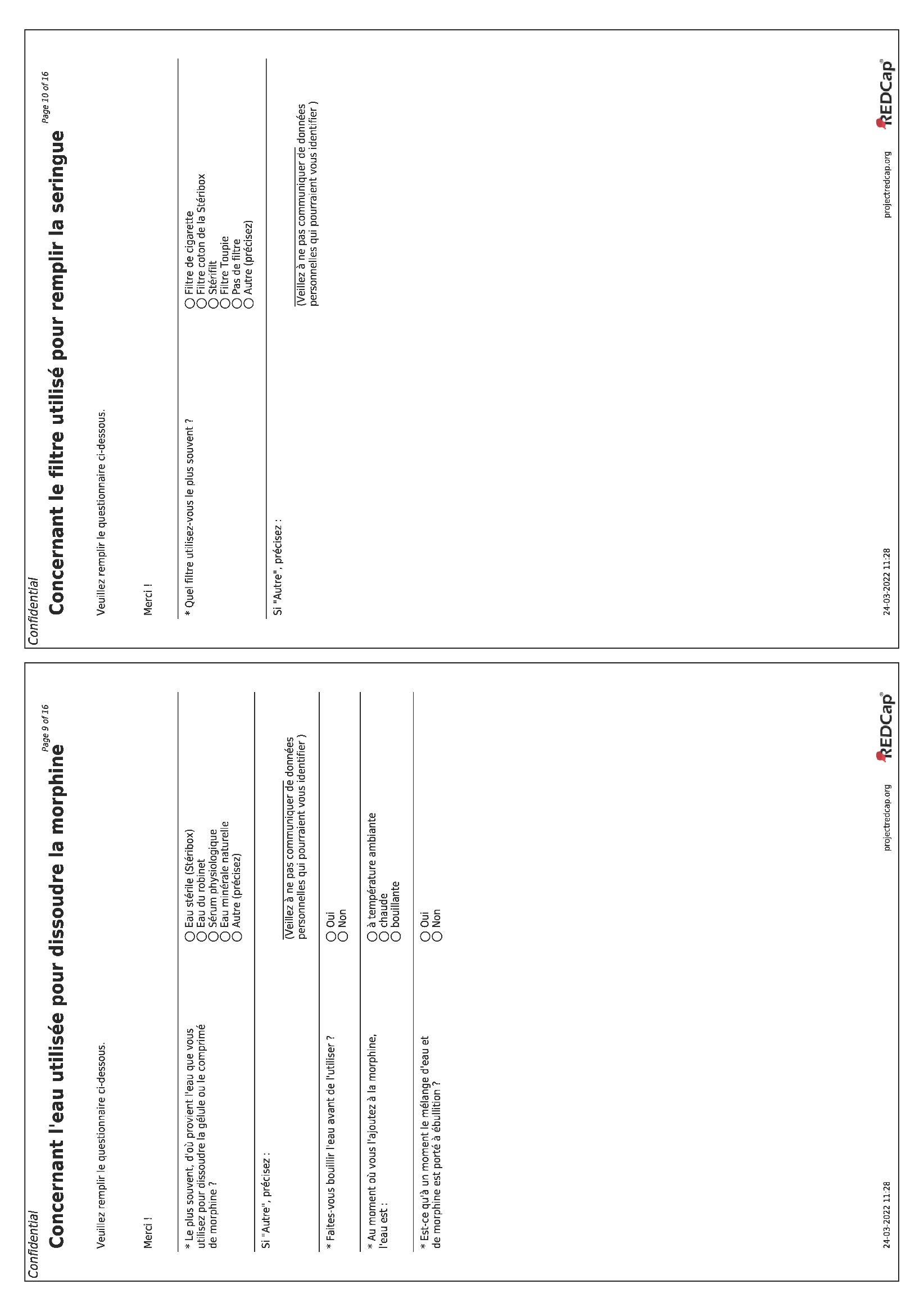


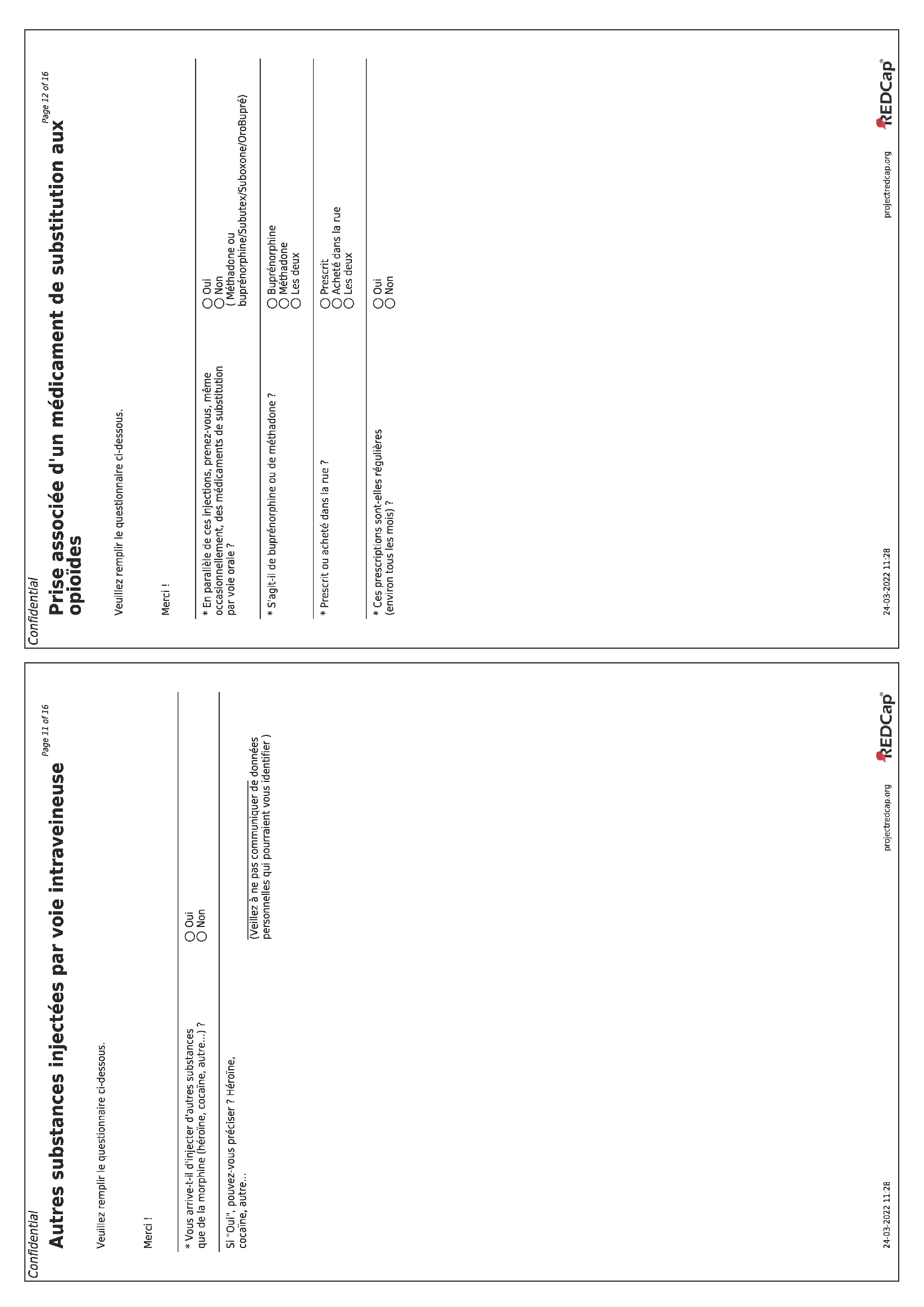

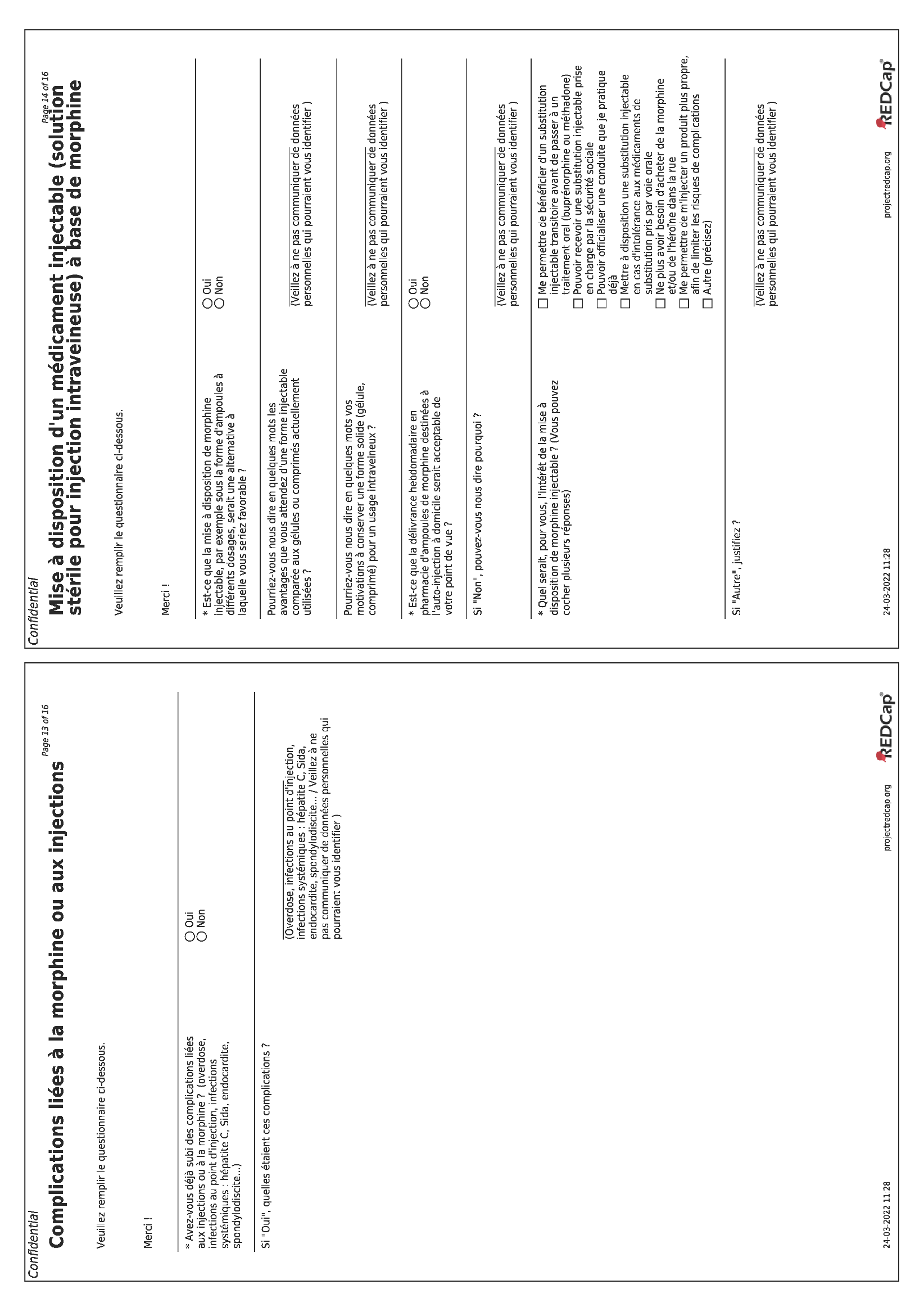


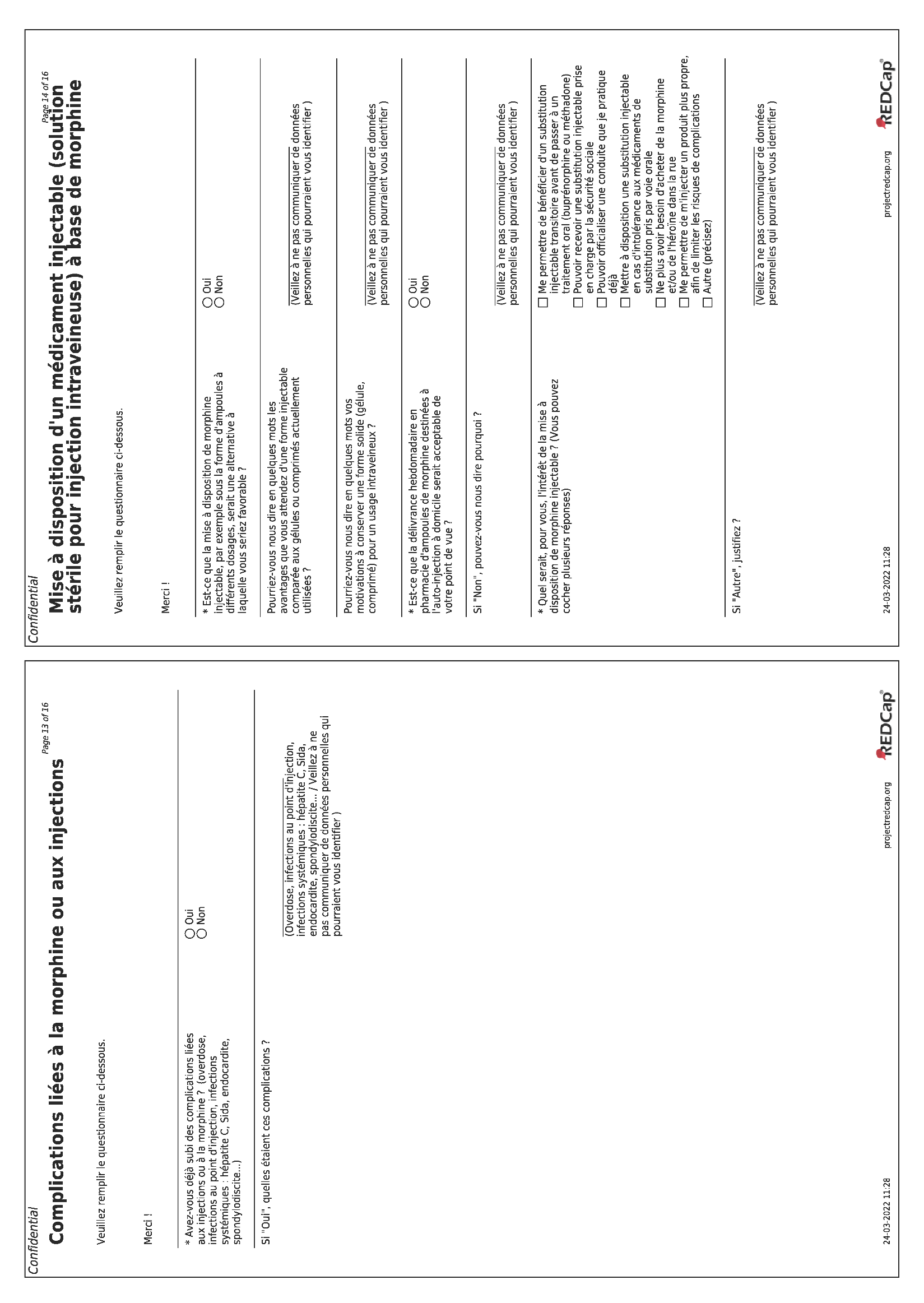


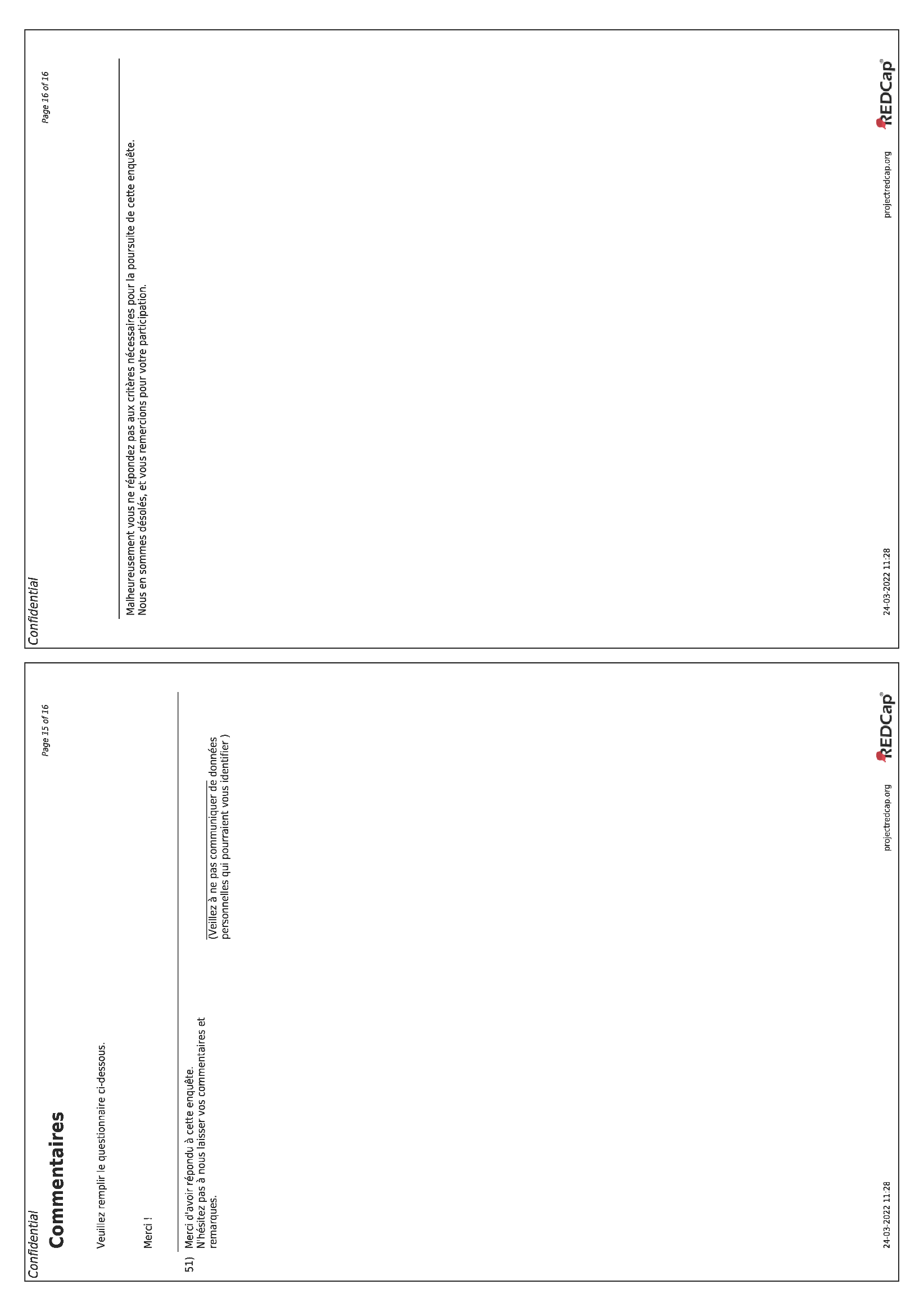


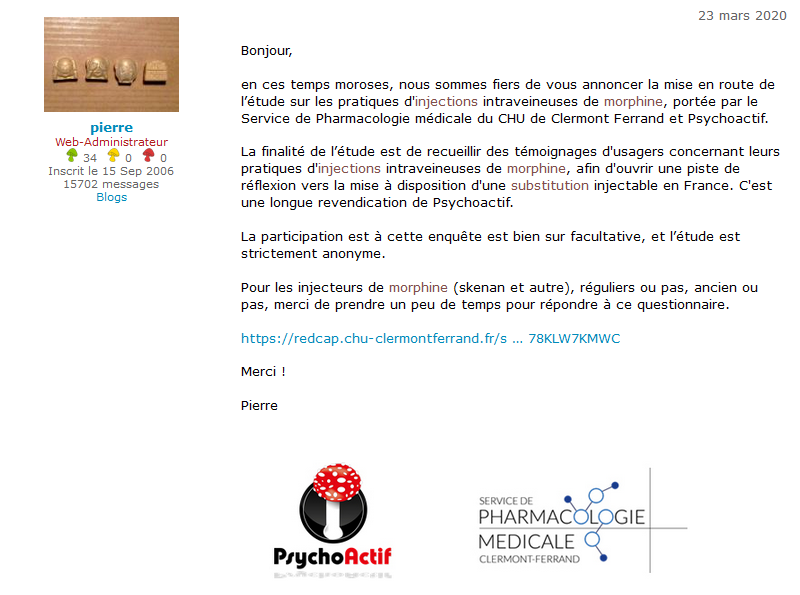


**Figure S2.** Survey presentation message on “PsychoACTIF” forum, in original language


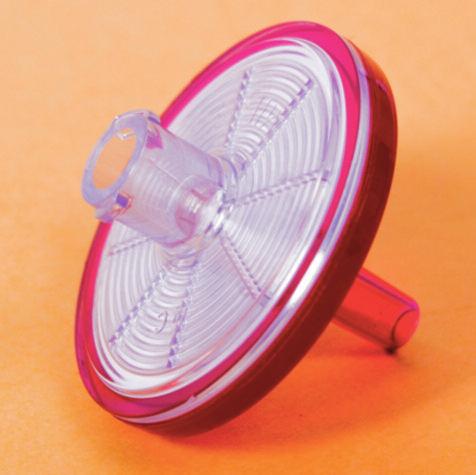

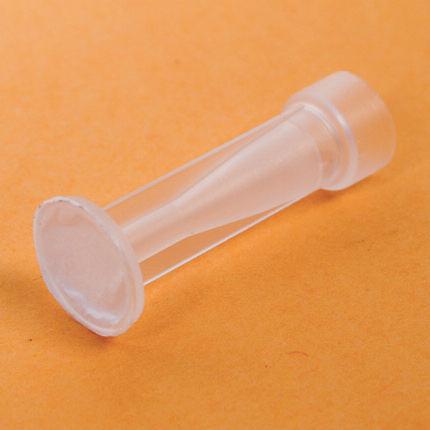

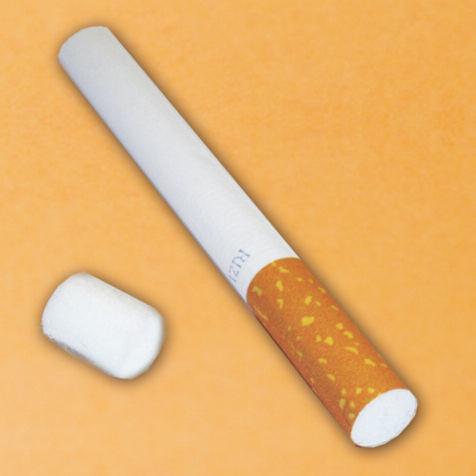


**Figure S5.** Filters used by users

From left to right: "Spinning top" harm reduction filter, Stérifilt^®^ harm reduction filter, cotton provided in the harm reduction kit Stéribox^®^, cigarette filter

Credit: psychoactif.org

**Figure S4.** Needles used by users, compared to their expectations


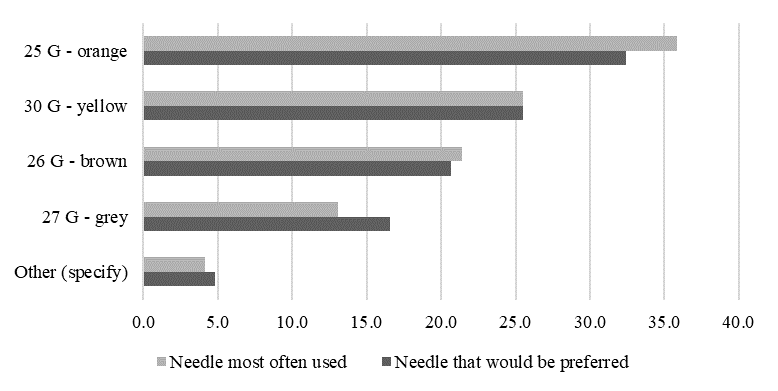


**Figure S3.** Syringes used by intravenous morphine users, compared to their expectations


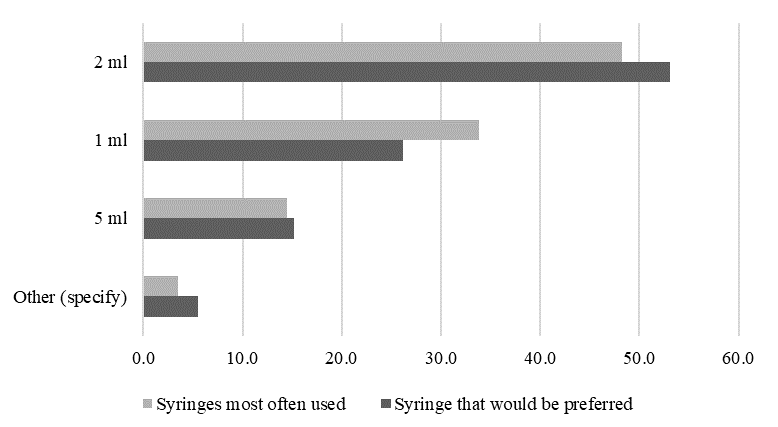

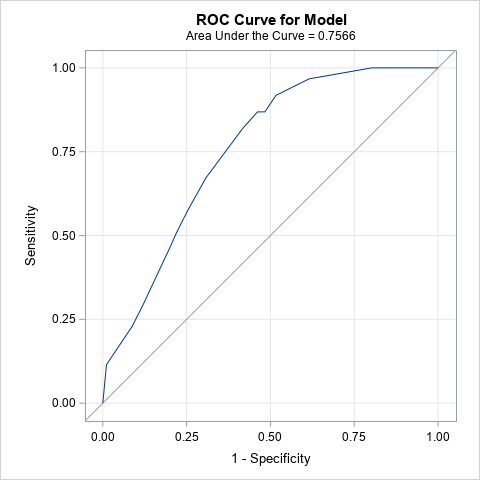


**Figure** **S6.** ROC Curve for multivariate analysis comparing people who never receive a prescription for their morphine to those who receive it regularly or not.

| **Table S1.** Results related to preparation methods | | |
| --- | --- | --- |
|  | Frequency  (Number) | Percent |
| **What kind of syringe do you use for your injections?** | | |
| 2 ml | 70 | 48.3 |
| 1 ml | 49 | 33.8 |
| 5 ml | 21 | 14.5 |
| Other (specify) | 5 | 3.5 |
| *(Frequency Missing = 13)* | | |
| **Ideally, what kind of syringe would you prefer to use?** | | |
| 2 ml | 77 | 53.1 |
| 1 ml | 38 | 26.2 |
| 5 ml | 22 | 15.2 |
| Other (specify) | 8 | 5.5 |
| *(Frequency Missing = 13)* | | |
| **What kind of needle do you use most often for your injections?** | | |
| 25 G – orange | 52 | 35.9 |
| 30 G – yellow | 37 | 25.5 |
| 26 G – Brown | 31 | 21.4 |
| 27 G – grey | 19 | 13.1 |
| Other (specify) | 6 | 4.1 |
| *(Frequency Missing = 13)* | | |
| **Idéalement, quel type d’aiguille préféreriez-vous utiliser ?** | | |
| 25 G – orange | 47 | 32.4 |
| 30 G – yellow | 37 | 25.5 |
| 26 G – Brown | 30 | 20.7 |
| 27 G – grey | 24 | 16.6 |
| Other (specify) | 7 | 4.8 |
| *(Frequency Missing = 13)* | | |
| **Where does the water you use to dissolve the morphine capsule or tablet usually come from?** | | |
| Sterile water (Steribox) | 111 | 76.6 |
| Tap water | 15 | 10.3 |
| Physiological serum | 11 | 7.6 |
| Natural mineral water | 5 | 3.5 |
| Other (specify) | 3 | 2.1 |
| *(Frequency Missing = 13)* | | |
| **Do you boil the water before using it?** | | |
| No | 90 | 62.1 |
| Yes | 55 | 37.9 |
| *(Frequency Missing = 13)* | | |
| **When you add it to the morphine, the water is:** | | |
| At room temperature | 79 | 54.5 |
| Hot | 56 | 38.6 |
| Boiling | 10 | 6.9 |
| *(Frequency Missing = 13)* | | |
| **Is the mixture of water and morphine brought to a boil at any point?** | | |
| No | 108 | 74.5 |
| Yes | 37 | 25.5 |
| *(Frequency Missing = 13)* | | |
| **Which filter do you use most often?** | | |
| “Spinning top” filter | 43 | 29.7 |
| Cotton filter (from Steribox) | 36 | 24.8 |
| Sterifilt | 36 | 24.8 |
| Cigarette filter | 19 | 13.1 |
| No filter | 7 | 4.8 |
| Other (specify) | 4 | 2.8 |
| *(Frequency Missing = 13)* | | |

| **Table S2.** Summary of respondents' answers to the question "If Yes, what were these complications?" | | |
| --- | --- | --- |
|  | Frequency  (Number) | Percent |
| Local bacterial infection (abscess) | 32 | 48.5 |
| Overdose | 19 | 28.8 |
| Hepatitis C virus | 15 | 22.7 |
| Systemic bacterial infection (Cotton fever, endocarditis, septic shock) | 9 | 13.6 |
| Thrombosis, pulmonary embolism | 7 | 10.6 |
| Veinitis | 4 | 6.1 |
| Human immunodeficiency virus | 1 | 1.5 |

| **Table S3.** Results of univariate analysis comparing people who never receive a prescription for their morphine to those who receive it regularly or not. | | | |
| --- | --- | --- | --- |
| **Associated factors** | **OR** | **95%CI** | **P (Wald)** |
| **Age** | | | <.01 |
| <25 *Versus >* 45 years | 4.0 | [1.3-12.6] |  |
| 25-34 *Versus >* 45 years | 2.0 | [0.7-5.9] |  |
| 35-45 *Versus >* 45 years | 0.9 | [0.2-2.6] |  |
| **Gender** | | | 0.6 |
| Male *Versus* Femelle | 1.3 | [0.5-2.9] |  |
| **Do you inject morphine regularly (several times a week or daily) or occasionally?** | | | <.01 |
| Occasionally *Versus* Regularly | 3.2 | [1.6-6.3] |  |
| **Is intravenous injection your most common route of administration?** | | | 0.3 |
| No *Versus* Yes | 1.6 | [0.7-3.4] |  |
| **Do you ever take any of your morphine by oral route?** | | | 0.1 |
| No *Versus* Yes | 1.8 | [0.9-3.7] |  |
| **Which of the following morphine-based medications do you use most often?** | | | 0.9 |
| Skenan *Versus* Other | 1.0 | [0.5-2.4] |  |
| **Do you ever inject substances other than morphine (heroin, cocaine, other...)?** | | | 0.3 |
| No *Versus* Yes | 1.5 | [0.7-3.5] |  |
| **In addition to these injections, do you take, even occasionally, oral substitution medication?** | | | 0.8 |
| Yes *Versus* No | 1.1 | [0.6-2.2] |  |
| **Have you ever experienced complications related to injections or morphine? (Overdose, injection site infections, systemic infections: hepatitis C, AIDS, endocarditis, spondylodiscitis...)** | | | 0.09 |
| No *Versus* Yes | 1.8 | [0.9-3.5] |  |
| **Would the availability of injectable morphine, for example in the form of vials of different dosages, be an alternative that you would accept?** | | | 0.5 |
| Yes *Versus* No | 1.7 | [0.3-9.2] |  |
| **Would weekly pharmacy delivery of morphine vials for home self-injection be acceptable from your perspective?** | | | 0.5 |
| Yes *Versus* No | 1.6 | [0.4-6.6] |  |
